# Testing a Real-Time Tenofovir Urine Adherence Assay for Monitoring and Providing Feedback to Preexposure Prophylaxis in Kenya (PUMA): Protocol for a Pilot Randomized Controlled Trial

**DOI:** 10.2196/15029

**Published:** 2020-04-02

**Authors:** Paul Drain, Kenneth Ngure, Nelly Mugo, Matthew Spinelli, Purba Chatterjee, Peter Bacchetti, David Glidden, Jared Baeten, Monica Gandhi

**Affiliations:** 1 University of Washington Seattle, WA United States; 2 Jomo Kenyatta University of Agriculture and Technology Nairobi Kenya; 3 Kenya Medical Research Institute Nairobi Kenya; 4 University of California San Francisco, CA United States

**Keywords:** PrEP, adherence, real-time monitoring and feedback, point of care, trial, Kenya, women, tenofovir, urine test, immunoassay

## Abstract

**Background:**

The worldwide expansion of preexposure prophylaxis (PrEP) with oral tenofovir-disoproxil-fumarate/emtricitabine will be critical to ending the HIV epidemic. However, maintaining daily adherence to PrEP can be difficult, and the accuracy of self-reported adherence is often limited by social desirability bias. Pharmacologic adherence monitoring (measuring drug levels in a biomatrix) has been critical to interpreting PrEP trials, but testing usually requires expensive equipment and skilled personnel. We have recently developed a point-of-care (POC) immunoassay to measure tenofovir in urine, allowing real-time adherence monitoring for the first time.

**Objective:**

The goal of this study is to examine a point-of-care adherence metric in PrEP to support and increase adherence via a randomized controlled trial.

**Methods:**

The paper describes the protocol for a pilot randomized controlled trial to test the acceptability, feasibility, and impact on long-term adherence of implementing a POC urine test to provide real-time adherence feedback among women on PrEP. Eligible women (n=100) will be HIV-negative, ≥18 years old, and recruited from a clinic in Kenya that provides PrEP. Participants will be randomized 1:1 to the intervention of providing real-time feedback via the assay versus standard of care adherence counseling. Acceptability by participants will be assessed by a quantitative survey, as well as by qualitative data collected via in-depth interviews (n=20) and focus group discussions (n=4 groups, 5-10 women each). Feasibility will be assessed by the proportion of women retained in the study, the mean number of missed visits, the proportion of planned urine assessments completed, and messages delivered, while in-depth interviews with providers (n=8) will explore the ease of administering the urine test. Tenofovir levels in hair will serve as long-term adherence metrics. A linear mixed-effects model will estimate the effect of the intervention versus standard of care on logarithmically transformed levels of tenofovir in hair.

**Results:**

This study has been funded by the National Institute of Health, approved by the Kenya Medical Research Institute Institutional Review Board, and will commence in June 2020.

**Conclusions:**

A novel urine assay to measure and deliver information on adherence to PrEP in real-time will be tested for the first time in this trial planned among women on PrEP in Kenya. Study findings will inform a larger-scale trial assessing the impact of real-time adherence monitoring/feedback on HIV prevention. Improving adherence to PrEP will have long-term implications for efforts to end the HIV epidemic worldwide.

**Trial Registration:**

ClinicalTrials.gov NCT03935464; https://clinicaltrials.gov/ct2/show/NCT03935464

**International Registered Report Identifier (IRRID):**

PRR1-10.2196/15029

## Introduction

The worldwide expansion of preexposure prophylaxis (PrEP) with oral tenofovir (TFV) disoproxil fumarate/emtricitabine (TDF/FTC) will be critical to ending the HIV epidemic. Oral PrEP has been recommended by the Centers for Disease Control and Prevention [1] and the World Health Organization [2], and is being implemented worldwide. During the early phases of implementation, there have been several challenges and lessons. Importantly, PrEP is only effective for those who are adherent [3], and maintaining daily adherence for prevention can be challenging [4,5].

Daily adherence to PrEP can be difficult to sustain. PrEP was effective in placebo-controlled trials among men-who-have-sex-with-men and transgender women [6], among intravenous drug users [7], and among both men and women in serodiscordant couples [8-10]. However, there was no efficacy of oral PrEP observed in two large trials conducted among young, sexually active women in Africa who were not in serodiscordant relationships [4,5]. In these studies, women in both trials reported >95% adherence to the study drug, but random plasma tenofovir levels among women on the active drug were detectable in fewer than one-third of participants [4,5].

Pharmacologic measures of PrEP drug predict the efficacy of PrEP more accurately than self-reported adherence [4,5,11-15]. In PrEP studies, studies have typically examined the predictive utility of drug concentrations retrospectively using biomatrices such as plasma [16], peripheral blood mononuclear cells [17], hair [18], and dried blood spots [19,20]. Drug levels are usually examined in these biomatrices via liquid chromatography/tandem mass spectrometry (LC-MS/MS). Real-time monitoring of PrEP drug levels, with direct feedback to clients, is difficult to do with LC-MS/MS testing due to the need for specialized equipment and laboratory-based personnel but may improve adherence to oral PrEP [21-24].

**Figure 1:**
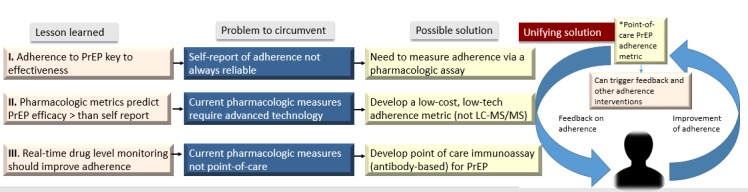
The rationale behind the PUMA study. PUMA: Point-of-Care Urine Monitoring of Adherence; PrEP: preexposure prophylaxis; LC-MS: liquid chromatography/tandem mass spectrometry.

Our team has developed a rapid point-of-care (POC) test to objectively assess TFV levels in urine as a measure for PrEP adherence [25]. The immunoassay is highly specific (100%), sensitive (96%), and provided TFV levels in urine that correlated strongly with LC-MS/MS-measured levels (r=0.95) in a study of volunteers administered daily TDF/FTC [25]. In a larger study, where TDF/FTC was administered to HIV-noninfected volunteers at 2, 4, and 7 doses a week, the assay showed the same excellent performance characteristics. From that, we were able to determine an adherence cut-off (in nanograms/milliliter) for TFV in urine for the point-of-care lateral flow assay [26]. We have also shown that low urine TFV levels by this immunoassay predicted future HIV seroconversion events in a large, completed, PrEP demonstration project. [27]. However, this tool has not yet been tested among people on PrEP to determine if real-time monitoring of adherence using a urine assay is feasible, acceptable, and improves PrEP adherence. The principles behind proposing such a trial are summarized in Figure 1.

The objective of this study is to perform a pilot randomized trial in Kenya to test the acceptability, feasibility, and impact on long-term adherence of implementing POC urine TFV testing and providing real-time feedback among women receiving PrEP. We will also conduct a sequential explanatory mixed-methods study to understand both user and provider experiences, preferences, barriers, and facilitators related to POC urine TFV adherence testing after the pilot trial. Our central hypothesis is that real-time PrEP adherence monitoring and feedback, now possible for the first time via a novel point-of-care adherence metric, will motivate adherence and eventually improve the preventative efficacy of PrEP.

## Methods

### Study Design

**Figure 2:**
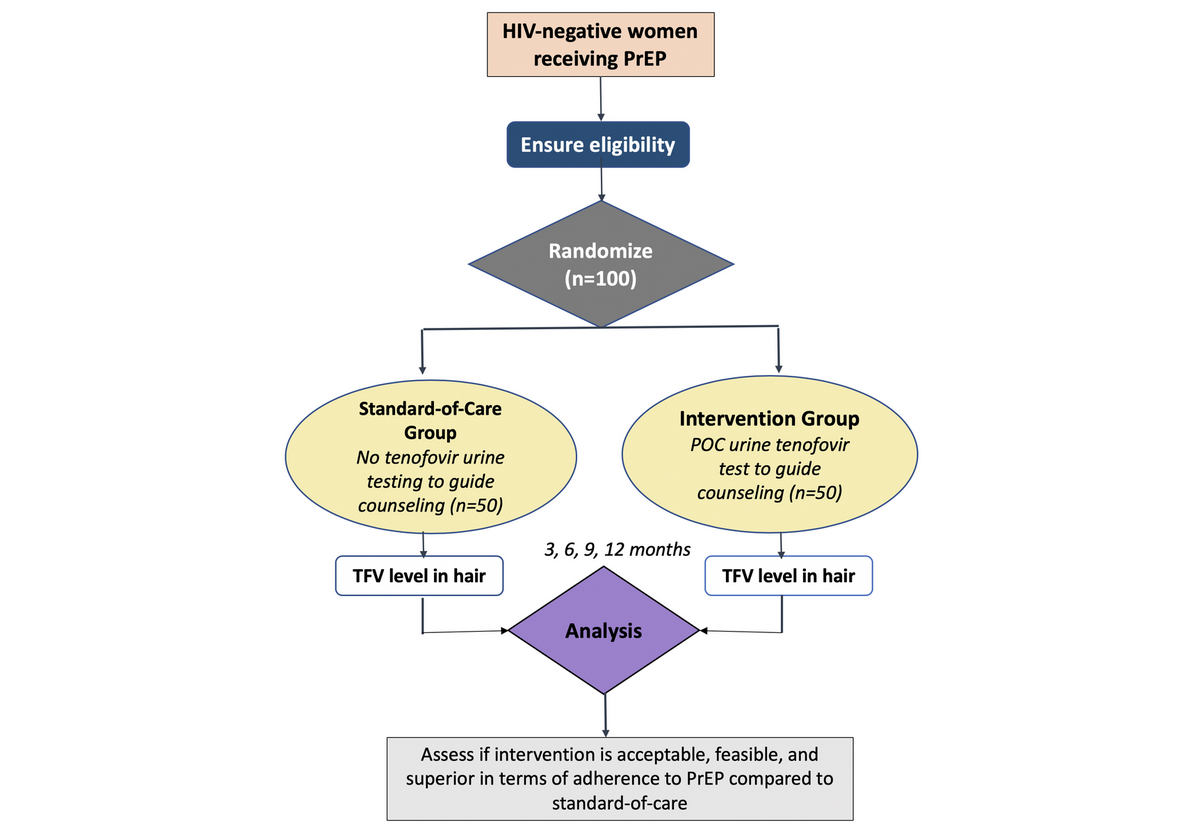
Schema for the PUMA study. PUMA: Point-of-Care Urine Monitoring of Adherence; PrEP: preexposure prophylaxis; TFV: Tenofovir.

The PUMA (Point-of-Care Urine Monitoring of Adherence) study is an open-label, 12-month, randomized controlled trial in healthy adult women receiving oral PrEP (TDF/FTC). The women receiving PrEP will be randomized (1:1) to the standard of care (n=50) versus POC urine assay testing (n=50) performed with real-time adherence feedback. Figure 2 describes the study design and the randomization process. Upon randomization, study visits will occur at 3, 6, 9, and 12 months after PrEP initiation.

### Human Subjects and Informed Consent

The study was filed for ethical approval with the Kenya Medical Research Institute Center for Clinical Research Scientific Committee Meeting (KEMRI/SERU/CCR/0123) and the University of California, San Francisco, Institutional Review Board. All participants will be screened for eligibility and asked to provide written informed consent before study participation. Patients can withdraw from the study at any time or will be withdrawn if HIV antibody testing is positive or if incident HIV infection is detected.

### Study Population

A total of 100 women will be recruited, with 50 randomized to the intervention arm and 50 to the standard of care arm (Figure 2). Eligible participants will be women who are HIV-negative, not in a serodiscordant relationship, have an estimated creatinine clearance >60 mL/min, are ≥18 years old, are receiving PrEP, and are returning for a follow-up visit three months after PrEP initiation (which is the second follow-up visit under Kenyan guidelines, with the first occurring at one month after initiation) [28]. We anticipate that randomizing women at their second PrEP follow-up visit, which occurs at three months after PrEP initiation, will help minimize attrition during the study. All people must be able and willing to provide informed consent to participate in the study.

### Study Location

The clinical study is being conducted at the Thika Clinic, which is located in an urban center about 40 kilometers outside of Nairobi, Kenya. The clinic is a center of excellence for PrEP delivery in Kenya.

### Recruitment, Enrollment, and Randomization

The Thika Clinic has established local recruitment and screening methods that operationalize protocol-specified requirements for eligibility determination in a manner that is tailored to and most efficient for the local setting and target population. Recruitment strategies will include partnering with existing voluntary counseling and testing centers, outreach workers, community organizations (eg, churches), and community mobilization around women’s voluntary counseling and testing promotion. Recruitment materials will educate women about PrEP. Screening and enrollment may occur on the same day or may be split across days, depending on the preference of the potential participant.

A member of the study team will approach individuals receiving PrEP. They will describe the study and ask for voluntary participation. Under the 2016 Kenya PrEP guidelines, all people initiating PrEP undergo a clinical assessment to ask about symptoms of acute HIV infection and receive rapid HIV and creatinine testing. Those who want to participate voluntarily will be taken to a private area of the clinic to be asked several demographic and clinical questions.

After obtaining written informed consent, participants will be randomized (1:1) to receive either POC urine TFV testing with same-day counseling or standard-of-care self-reported adherence monitoring (Figure 2). The study statistician will generate an allocation sequence with random numbers using SAS 9.4 (SAS Institute Inc, Cary, North Carolina, United States). Sequentially numbered, sealed, opaque envelopes containing a study arm allocation and participant identification number will be opened once an eligible, consenting participant is enrolled.

### Study Procedures

**Figure 3:**
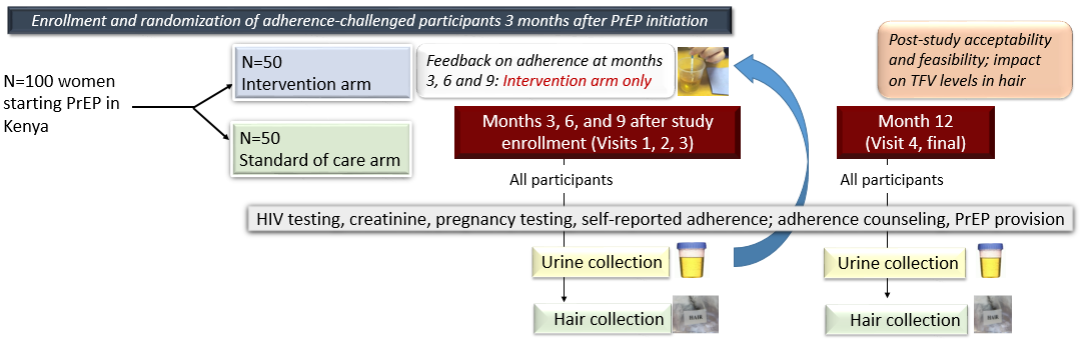
Schedule of evaluations for participants in the intervention and standard of care arms for the PUMA study. PUMA: Point-of-Care Urine Monitoring of Adherence; PrEP: preexposure prophylaxis; TFV: Tenofovir.

The study will follow all aspects of Kenya’s PrEP guidelines [28] except that those randomized to the intervention arm will receive POC adherence testing by a urine TFV assay (Figure 3). Once written consent is obtained, a member of the study team will collect demographic and health questions related to age, birthdate, income, employment history, prior HIV testing, medical conditions, and current symptoms. They will also obtain each participant’s phone number, address, and relevant contact information. After obtaining baseline demographic and clinical data, the study group assignment of the participant will be determined by a random process. A research nurse will then meet the participant in the same clinical exam room and administer a brief clinical questionnaire. The nurse will coordinate the necessary blood draws for the participant so that each participant will only have one blood draw for each clinical visit.

People who qualify for and initiate PrEP in Kenya according to the 2016 Kenyan PrEP guidelines [28] are subsequently seen in one month, in three months, and then every three months for repeat HIV testing. Patients on PrEP undergo continued risk assessment and adherence counseling for daily PrEP under the standard of care. PrEP delivery includes measurement of renal function (estimated creatinine clearance >60 mL/min to start PrEP and periodic monitoring over time, aligned to the visit schedule of the study), standard clinical assessment to avoid the continuation of PrEP during acute HIV infection, and adherence counseling. Participants in both arms will be counseled on PrEP and provided with three months of PrEP medication after the one-month visit, according to Kenyan national guidelines.

### Follow-Up Visits

At study baseline and months 3, 6, 9, and 12 after enrollment, the study team will collect urine, plasma, whole blood from dried blood spots, and hair samples. Table 1 provides a summary of clinical visits and testing in both study arms. At each study visit, we will offer counseling for participants for HIV testing (pre- and posttesting), HIV infection risk reduction best practices, condom promotion and provision, adherence to PrEP medication, as well as other HIV prevention strategies. Participants in the standard-of-care arm will be directed to the regular clinical waiting area to be seen and evaluated by the study team every three months until study end (Month 12). The study team will prescribe PrEP and additional medications, as well as provide adherence counseling, as appropriate. Kenyan guidelines recommend conducting rapid testing for HIV before dispensing PrEP at each visit. If any participants have a positive rapid test by an oral or blood-based test, confirmatory testing will then be performed, and participants will receive standard HIV care, including initiation of antiretroviral therapy. All participants will receive HIV counseling, condoms, risk reduction counseling, and syndromic management of sexually transmitted infections according to local guidelines.

**Table 1:** Summary of the clinical visits and laboratory testing for study groups.

Visit requirements			Enrollment		Month 3		Month 6		Month 9		Month 12
**Screening and enrollment**											
	Review eligibility criteria	✓		—		—^a^		—		—	
	Obtain informed consent	✓		—		—		—		—	
	Randomization	✓		—		—		—		—	
	Collect sociodemographic information	✓		—		—		—		—	
**Research assistant tasks**											
	Collect and update contact information	✓		✓		✓		✓		✓	
	Conduct baseline questionnaire	✓		—		—		—		—	
	Conduct quarterly questionnaire	—		✓		✓		✓		—	
	Conduct exit study questionnaire	—		—		—		—		✓	
**Clinical visit by nurse or physician**											
	Medical history and interval updates	✓		✓		✓		✓		✓	
	Physical examination (as needed)	✓		✓		✓		✓		✓	
	Rapid HIV testing	✓		✓		✓		✓		✓	
	PrEP^a^ or drug side effect screen	✓		✓		✓		✓		✓	
	Collect/assess pill count for prior PrEP	✓		✓		✓		✓		✓	
	PrEP dispensing for 3-month supply	✓		✓		✓		✓		✓	
	Adherence and risk reduction counseling	✓		✓		✓		✓		✓	
**Point-of-care and laboratory testing**											
	Serum hemoglobin	✓		—		—		—		—	
	Serum creatinine	✓		—		✓		—		✓	
	Urinalysis	✓		—		✓		—		✓	
	POC^b^ urine TFV^c^ assay (intervention arm)	✓		✓		✓		✓		✓	
	POC urine TFV assay (standard-of-care arm)	—		—		—		—		✓	
**Specimen collection and storage**											
	Stored plasma	✓		✓		✓		✓		✓	
	Dried blood spot for TFV-DP^d^	✓		✓		✓		✓		✓	
	Hair sample for TFV-DP	✓		✓		✓		✓		✓	

^a^Not applicable.

^b^PrEP: preexposure prophylaxis.

^c^POC: point-of-care.

^d^TFV: tenofovir.

^e^TFV-DP: tenofovir-diphosphate.

**Figure 4:**
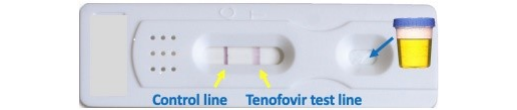
Prototype of point-of-care urine-based tenofovir assay.

Participants in the intervention arm will receive the same treatment as participants in the standard-of-care arm, and also quarterly testing for TFV by a rapid urine diagnostic test. The results of the test will be provided to the participant and will be used to inform enhanced adherence counseling for those who do not demonstrate adequate PrEP adherence. An image of the POC urine adherence assay is shown in Figure 4.

Subsequently, clinic-based POC testing for urine TFV will occur at quarterly visits during the 12-month study period. Counseling messages to the women in the intervention arm will be delivered by the study team, which includes staff with extensive experience in counseling and behavioral interventions. The messages to provide feedback on adherence will be adapted from the HPTN082 (HIV Prevention Trials Network 082) study and other studies that have provided PrEP pharmacologic feedback [29]. The counseling messages for this trial will first be piloted in a small group of women on PrEP at Thika for refinement before actual implementation.

### Study Outcomes

Participants enrolled in this study will be followed for a total of 12-months from the date of enrollment. The three primary outcomes for this study will be: (1) feasibility of the intervention; (2) acceptability of POC urine tenofovir testing among women receiving PrEP; and (3) preliminary impact on adherence as assessed by a long-term metric of adherence (eg, hair levels).

#### Feasibility

The feasibility of this intervention will be assessed by the proportion of women retained in the study at 12 months, the mean number of missed visits, the proportion of planned urine assessments completed, and the proportion of messages delivered in the intervention arm. Qualitative data on feasibility will also be assessed via in-depth interviews with research providers at the clinic (n=8) on the acceptability and ease of administering the urine POC test. We will also assess PrEP refills, HIV testing completion, and safety (including accuracy of HIV testing, management of side effects, and social harm). We will also establish via the interviews with providers whether they liked the yes/no assay or would prefer an assay that has more lines indicating high, moderate, or low adherence.

#### Acceptability

Acceptability will be assessed by a quantitative survey of participants at the end of the study (n=50 in the intervention arm) as well as via qualitative data collected via in-depth interviews with participants (n=20) and focus group discussions (n=4 groups; 5-10 women each). Items to be assessed in the quantitative surveys include questions on feelings about receiving their PrEP adherence results in real-time, likelihood of wanting to receive results of urine testing outside of a study while they are on PrEP, concern about the privacy and security of the data regarding their urine results, grading of the potential impact of knowing their urine TFV results on subsequent medication adherence, and likelihood of taking PrEP just before study visits because they knew the urine test was being conducted. The semistructured interview guide for the qualitative interviews will elicit feelings about the adherence metric and counseling messages, concerns regarding privacy, advantages/disadvantages of receiving such results, and the likely impact of this monitoring test on sustained adherence to PrEP or just short-term adherence. Finally, we will also establish via the interviews whether women liked the yes/no assay or would prefer an assay that has more lines indicating high, moderate, or low adherence.

#### Long-Term Adherence

Hair levels of TFV and FTC in hair samples will be measured at the 0 (baseline), 3, 6, 9, and 12-month clinic visits after enrollment in the study [18,30,31]. Drug concentrations will be measured at the Hair Analytical Laboratory at the University of California, San Francisco, using validated LC-MS/MS assays [18]. Our methods have been peer-reviewed and approved by the Division of AIDS’ Clinical Pharmacology Quality Assurance and Quality Control Program [32], which is based on the US Food and Drug Administration’s Guidance for Industry Bioanalytical Method Validation. Hair levels will serve as the efficacy outcome of the pilot trial. Of note, incident HIV infection will be measured but is expected to be low given that individuals will be taking PrEP, and the study would need to be considerably larger to assess incident HIV with much precision. Finally, we will assess genotypic HIV resistance among any seroconverters.

### Sample Size Calculation

We assessed sample size based on the primary effectiveness outcome of this pilot randomized trial, an increase in hair levels with the real-time feedback/monitoring in the intervention arm compared to the standard of care arm. For the hair level outcome, we considered the simplified situation of estimating the difference in hair level changes between intervention and control arms using a single postintervention level from each person. In this scenario, and assuming the person-to-person variability in TFV hair levels observed in a similar population (women in Africa not in known, mutually-disclosed serodiscordant couples) in VOICE (Vaginal and Oral Interventions to Control the Epidemic) [5], the 95% confidence interval for an observed difference in means of log (TFV level) would extend 1.5-fold up and down from the estimate with 50 per arm. Given the 7- to 14-fold differences in median hair levels from different adherence levels in various PrEP studies [31], this precision is likely to provide strong enough evidence for improved adherence to justify a subsequent trial of efficacy for preventing HIV infection. Our actual precision will likely be better because of multiple observations per person and the contribution of within-person changes to the overall estimate.

### Statistical Analysis

The primary biostatisticians for the study and the Thika Clinic research staff will conduct data management by including procedures to ensure data quality, double data entry, range checks for data values, and encrypting data without patient identifiers for blinded analysis. Participant contact tracing for retention at month 6 and 12 will be performed, particularly to establish PrEP continuation and HIV status. We will use structured interviews on HIV testing practices and self-reported PrEP adherence (eg, frequency, ability, self-rating, missed doses). We will use REDCap (research electronic data capture) to record all study data.

Our primary analysis of the adherence outcome will be a linear mixed-effects model to estimate the effect of the intervention versus standard-of-care on logarithmically transformed levels of TFV in hair. Since large relative differences between very low hair levels are less clinically important than similar relative differences between higher levels, we will Winsorize [33] undetectable levels to equal the detection limit, and we will add the detection limit to all levels before log transformation. These steps reduce the influence of minor differences between levels at or near the detection limit while preserving the approximate interpretation of back-transformed regression coefficients as fold-effects. The predictor variable for intervention will equal zero for everyone at the baseline (month 0) visit (where hair levels reflect prerandomization baseline) and then will change for those in the intervention arm to equal one at all subsequent visits. A random intercept term will account for within-person correlation across multiple visits. The timepoint will be included as a categorical variable to account for systematic changes over time. We note that inclusion of the preintervention baseline levels will allow the model to account for some of the random person-to-person variation as well as allowing within-person changes to contribute to the estimate of the intervention’s effect. Correlations between self-reported adherence over 30 days and hair levels will also be calculated.

Analyses will be by intention-to-treat. Tenofovir levels in hair will be compared between arms using repeated measures mixed-effects models. PrEP discontinuation, defined as missing any refill, will be analyzed as a time-to-event outcome using Cox proportional hazards regression. If the treating clinician discontinues PrEP for safety reasons (but not adherence reasons), follow-up after that will be censored. Adjusted analyses will be done to control for potential confounders based on our prior work assessing correlates of PrEP use: demographics (eg, age, educational level), sexual behaviors (eg, condom use, outside partnerships), medical status (eg, depression), and beliefs (eg, risk perception, PrEP efficacy). SAS Software will be used for analyses.

## Results

This study was funded by the National Institutes of Health in September 2018 (R01AI143340) and approved by the Kenya Medical Research Institute Institutional Review Board in August 2019. The study is projected to start in June 2020, with data collection from June 2020 to January 2022, data analysis commencing January 2022, and publication expected by June 2022.

## Discussion

A point-of-care test to analyze tenofovir concentrations in urine has recently been developed, and this protocol paper describes the design of the first pilot clinical trial incorporating POC adherence testing and feedback for participants on PrEP. Given the critical contribution of adherence to the preventative efficacy of PrEP, novel interventions to improve long-term PrEP adherence will bolster the ability of this powerful preventative strategy to stem the tide of the HIV pandemic. Self-reported adherence to PrEP is limited by social desirability bias, and no surrogate of PrEP adherence (such as HIV viral loads in those living with HIV) exists. The field has, therefore, increasingly relied on pharmacologic metrics to objectively verify PrEP adherence, but the current methods to measure drug levels are expensive and laboratory-based. The development of a novel antibody-based POC test to monitor PrEP adherence in real-time could be transformative in how we document and intervene against poor PrEP adherence, but this trial will test that concept for the first time.

Prior studies have revealed that real-time monitoring and feedback on adherence could be motivating. In the VOICE study, a placebo-controlled randomized trial testing PrEP in African women, at least 50% of women on the active drug had undetectable TFV in all plasma tested. However, participants over-reported adherence, even at the last study visit where accurate reporting would not affect study participation [5]. Further qualitative work to understand nonadherence in the VOICE study was performed through the Microbicides Trials Network–003D study, which recruited participants with pharmacokinetic (PK) data consistent with low (0% of plasma samples having detectable TFV), inconsistent, or high adherence for retrospective disclosure of plasma TFV results and subsequent capture of reactions [21]. Women with low adherence first expressed surprise at the PK results, then acknowledged they were true and revealed reasons for non-drug-taking during in-depth interviews. Women in all three categories stated that real-time monitoring and feedback would improve adherence and that they would be more honest if presented with objective adherence metrics.

In the iPrEx (Iniciativa Profilaxis Pre-Exposición) open-label extension (OLE) study, a demonstration project of PrEP among men-who-have-sex-with-men and transgender women, drug level testing was performed in plasma and dried blood spots [22]. The results of plasma drug levels over prior weeks were shared with individuals at a later visit, which was reported as highly acceptable [23]. Those with detectable drug in their plasma appreciated receiving validation of adherence, and those without drug detection were not surprised. An in-depth qualitative analysis from iPrEx OLE confirmed the acceptability of drug level feedback and, for those who were not adherent, the motivating effect of receiving such feedback on subsequent adherence. The results of these interviews led authors to conclude that: (1) drug level feedback should be provided as quickly as possible, so new methods should be sought to provide rapid feedback; and (2) drug level feedback will encourage frank adherence discussion and should be provided in the context of adherence counseling.

Urine is a suitable matrix for POC testing for PrEP adherence since urine collection is noninvasive, preferred among youth over blood sampling, and TFV levels in urine correlate with TDF adherence [34-36]. Urine levels reflect plasma levels, which may be particularly useful when daily PrEP adherence is necessary [37]. Plasma and urine PrEP drug levels reflect only short-term exposure, and thus monitoring via these matrices may be susceptible to “white coat adherence,” where adherence improves transiently before a visit. Despite this theoretical concern, this phenomenon has rarely been observed in PrEP delivery [31].

At the study’s end, we will have conducted a field test of a novel immunoassay to quantitate TFV in urine as the first POC low-cost adherence metric available for participants on PrEP. The assay will launch a novel modality for adherence intervention. Moreover, the assay could be combined with digital technologies to support PrEP adherence, which are of burgeoning interest in the PrEP adherence intervention field [38,39], especially among youth [40]. Finally, the study findings will inform a larger-scale PrEP trial assessing the impact of real-time adherence monitoring/feedback via the assay. We expect this new TFV POC assay, the metabolite of both TDF and tenofovir alafenamide, to have widespread utility in both HIV prevention and treatment globally. The pilot trial proposed here will examine the acceptability, feasibility, and impact of this novel TFV adherence assay in urine for the first time. Improving adherence to both PrEP and ART will help reduce and prevent HIV transmission in the efforts to end the HIV/AIDS epidemic.

## Abbreviations

HPTN082: HIV Prevention Trials Network 082
iPrEx: Iniciativa Profilaxis Pre-Exposición
LC-MS/MS: liquid chromatography/tandem mass spectrometry
MTN: Microbicides Trials Network
OLE: open-label extension
POC: point of care
PrEP: preexposure prophylaxis
PK: pharmacokinetic
PUMA: Point-of-Care Urine Monitoring of Adherence
REDCap: Research Electronic Data Capture
TDF/FTC: tenofovir disoproxil fumarate/emtricitabine
TFV: tenofovir
VOICE: Vaginal and Oral Interventions to Control the Epidemic
